# Basal Cell Adenoma of the Minor Salivary Glands in the Buccal Mucosa: A Rare Entity Arising in an Unusual Location

**DOI:** 10.7759/cureus.36580

**Published:** 2023-03-23

**Authors:** Fayez A Alrohaimi, Farhan M Alanazi, Hisham M Almousa, Abdulaziz B Almutairi, Sultan M Alqahtani

**Affiliations:** 1 Otolaryngology - Head and Neck surgery, Prince Sultan Military Medical City, Riyadh, SAU; 2 Otolaryngology - Head and Neck Surgery, Prince Sultan Military Medical City, Riyadh, SAU; 3 Otolaryngology - Head and Neck Surgery, Prince Mohammed Medical City, Al Jouf, SAU; 4 Medicine, King Saud University, Riyadh, SAU; 5 Medicine, Majmaah University, Al Majmaah, SAU

**Keywords:** salivary gland tumors, painless lump, benign oral mass, buccal mucosa, minor salivary gland, basal cell adenoma

## Abstract

One of the rare tumors of the salivary gland is known as basal cell adenoma (BCA). Only a small percentage of salivary gland tumors affect the minor salivary gland of the oral cavity while the majority are found in the parotid gland. We present a rare case of BCA involving the left buccal mucosa of a 45-year-old female. Magnetic resonance imaging (MRI) showed well defined solid mass measuring 1.9 x 1.5 cm in the left buccal space inseparable from the buccinator muscle. The T2-weighted image demonstrates a hyperintense signal post-contrast. Ultrasound-guided fine needle aspiration cytology revealed cellular basaloid neoplasm of uncertain malignant potential. Thereafter excision of the mass was performed through a transoral approach under general anesthesia. Histopathology of the mass showed encapsulated basal cell neoplasm in favor of BCA. The patient was doing well after the surgery and has intact facial nerve and adjacent nerves such as the auriculotemporal nerve and great auricular nerve with no complications then she kept on routine clinic follow-ups, and the surgical site recovered successfully. Therefore, we conclude that MRI and biopsy provide useful information to differentiate between benign adenoma and malignant adenocarcinoma. BCA should be considered in a differential diagnosis of an isolated neck mass. Surgical excision demonstrates an excellent prognosis.

## Introduction

Salivary glands basal cell adenoma (BCA) is a benign tumor that is rare and has a high risk of recurrence with a generally good prognosis. It’s classified as a separate entity in the classification of tumors of the salivary glands by the World Health Organization (WHO) [1]. Several sites have been identified in the literature, with the parotid gland being the most frequently reported, followed by the upper lip, buccal mucosa, lower lip, and palate. BCA usually affects people between their fifth and seventh decades of life. Histologically, four different patterns have been described: solid, tubular, trabecular, and membranous [2-4]. Due to prognostic considerations, a list of differential diagnoses is required to distinguish between BCA and other tumors such as adenoid cystic carcinoma (ACC), basaloid squamous cell carcinoma (BSCC), and basal cell adenocarcinoma (BCAC) [5]. This case report includes a review of related findings as well as specifics of a BCA that begin in a minor salivary gland of the left buccal mucosa.

## Case presentation

**Figure 1:**
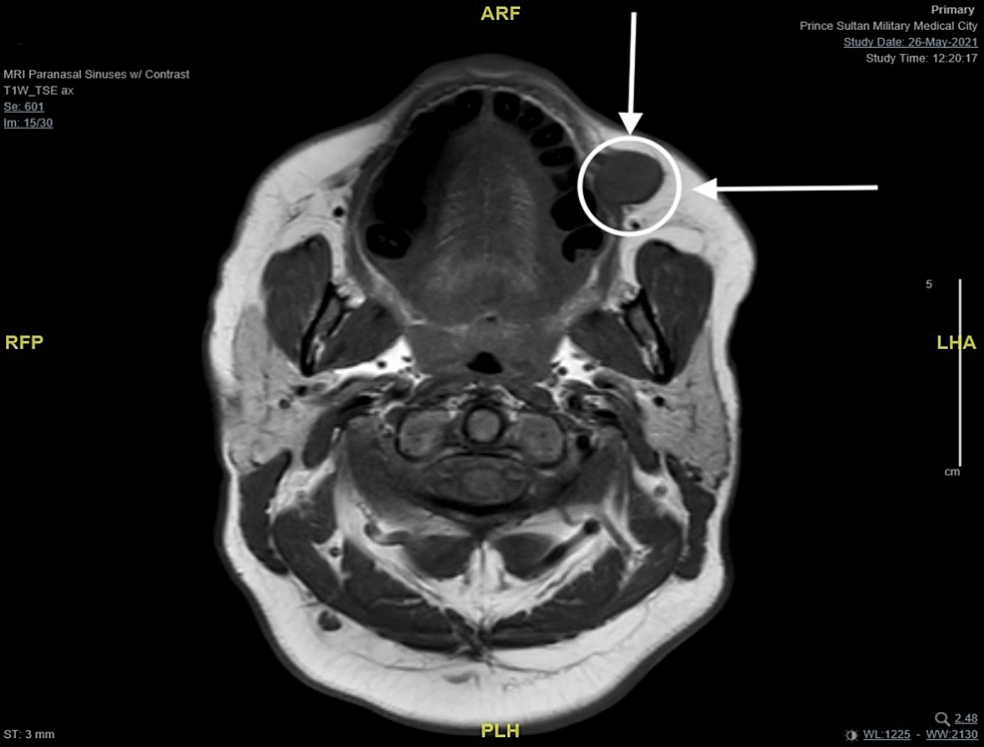
MRI T1-weighted image (axial cut) demonstrating iso-intense mass on the left buccal space. MRI: magnetic resonance imaging

**Figure 2:**
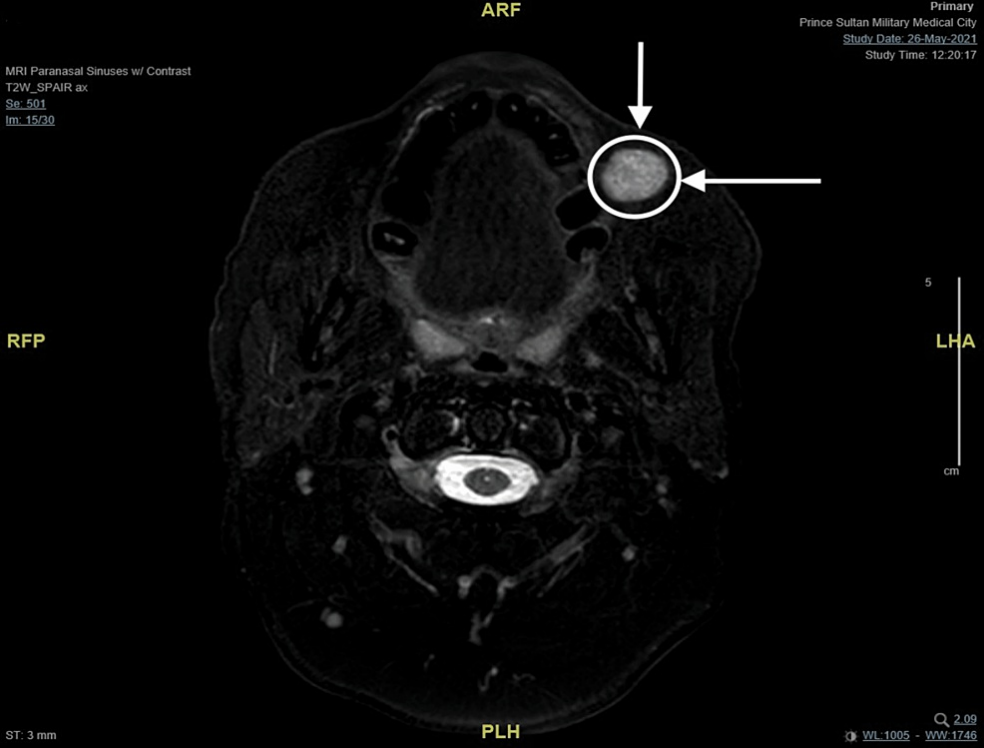
MRI T2-weighted image (axial cut) demonstrating hyperintense mass post contrast on the left buccal space. MRI: magnetic resonance imaging

**Figure 3:**
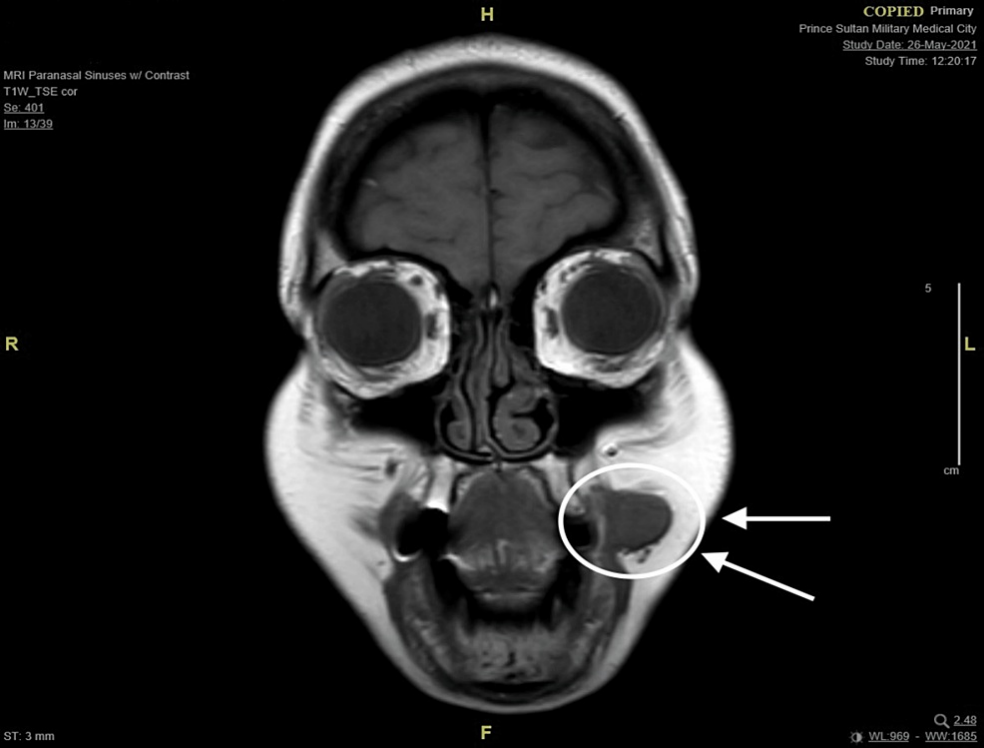
MRI T1-weighted image (coronal cut) demonstrating iso-intense mass on the left buccal space. MRI: magnetic resonance imaging

**Figure 4:**
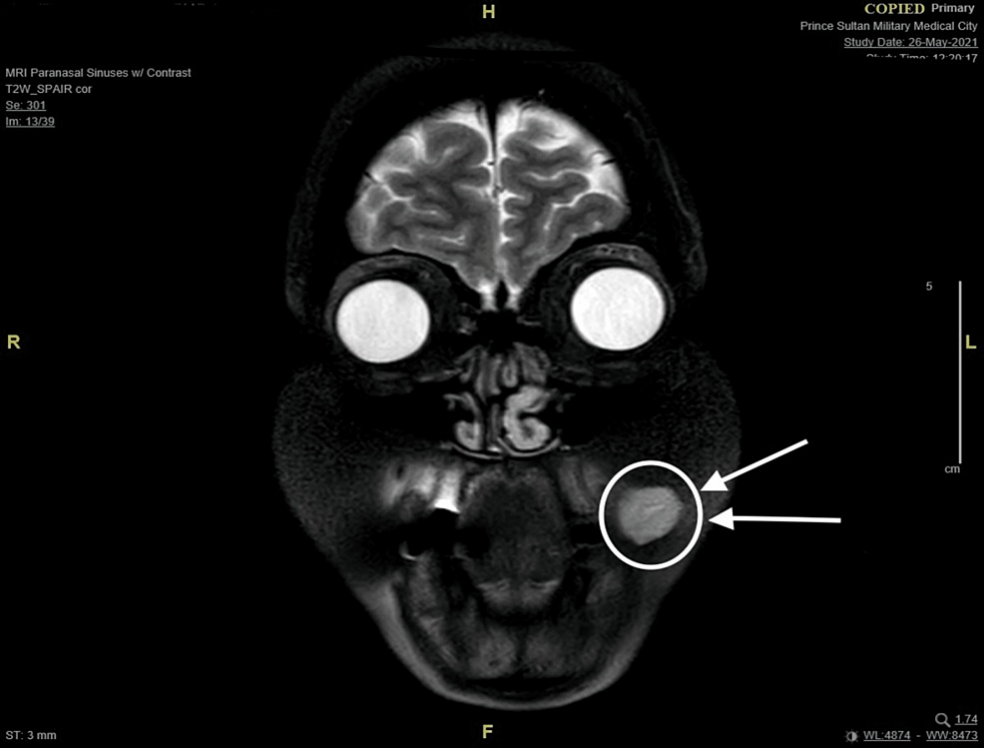
MRI T2-weighted image (coronal cut) demonstrating hyperintense mass post contrast on the left buccal space. MRI: magnetic resonance imaging

A 45-year-old female not known to have any medical illness was referred to the clinic to evaluate a painless, asymptomatic mass at the left buccal mucosa noticed seven years ago which was small and then had been slowly growing over the past five years. It started off being easily moveable and over time became more fixed. She denied any history of pain, skin discoloration, numbness, facial weakness, xerostomia, difficulty breathing, dysphagia, odynophagia, or other compressive symptoms. A bimanual examination of the oral cavity revealed an intraoral left buccal mass that is round, firm, mobile, and not attached to the skin with no fluctuations. There were no skin ulcers or palpable lymph nodes, and the rest of the head and neck evaluation was unremarkable. Ultrasound-guided fine needle aspiration cytology was performed on the patient, which demonstrated a cellular basaloid tumor with uncertain malignant potential. The head and neck magnetic resonance imaging (MRI) revealed a well-defined solid mass measuring 14x19x15mm (anterior-posterior, transverse, and craniocaudal diameter respectively) centered at the left buccal space. This lesion is showing an isointense signal on T1, an intermediate signal on T2 with the internal vascular signal void, and homogeneous intense enhancement post-contrast with the impression of low-grade minor salivary gland tumor (Figures 1-4). After reviewing several treatment options for such masses with the patient and the benefits of surgery, the patient agreed to have a left buccal mass excision through a transoral approach. She was doing well on the first day after surgery, with a clean wound and intact facial nerve and adjacent nerves such as the auriculotemporal nerve and great auricular nerve with no complication then she was discharged home the day after the procedure in good stable condition. Histopathology report of the mass showed encapsulated basal cell neoplasm in favor of BCA. The patient was kept on routine clinic follow-ups, and the surgical site recovered successfully.

## Discussion

The worldwide histological classification of tumors was created by the World Health Organization (WHO) in 1972, adenomas of the salivary gland were categorized into two types: monomorphic and pleomorphic adenomas. BCA was classified as a form of monomorphic adenoma in the first version published in 1972 [6]. Furthermore, in 1991 the second version was published, and they classify salivary adenomas into nine different types, in this histological classification BCA was included [1]. BCA was found in less than 3% of all tumors of the salivary glands, and around 75% of BCA is located in the parotid gland while the rest affect the minor salivary glands, particularly in the upper lip [7,8]. Since 1991 About 21 case reports of basal cell adenoma in the minor salivary glands, including our case, were reported in the literature when the tumor was identified as a separate salivary gland tumor [7-18]. In the 21 cases, the average age of the patients was 48.2 ± 12.0 years, and there was no gender preponderance. Most of the cases were found on the palate (seven case reports), upper lip (seven case reports), buccal mucosa (five case reports), and alveolus (two case reports). The average size of the reported cases was 2.5 ± 1.0 cm. However, in our case, it was 14x19x15mm (anterior-posterior, transverse, and craniocaudal diameter, respectively).

Histopathologically, BCA is composed of fairly monotonous and homogeneous cells that resemble basal cells and they are distinguished by the growth of a mass from these cells that resembles a tumor. Depending on the World Health Organization (WHO) classification, basal cell adenoma is categorized into four groups according to the way the adenoma is arranged: solid, tubular, trabecular, and membranous [1]. Nevertheless, there are some cases of mixed types that have been reported [19,20]. The tumor's Histopathology in the present case showed encapsulated basal cell neoplasm in favor of basal cell adenoma. Among the 21 cases described above, 13 had solid tumors, seven had trabecular tumors, nine had tubular tumors, and two had membrane tumors (with overlaps of mixed types), in our case it was cellular basaloid neoplasm of uncertain malignant potential. This finding supports the previous reports which state that solid tumor is the most common type [21].

Differentiation between basal cell adenocarcinoma (BCAC) from BCA sometimes is challenging. One of the diagnostic criteria to differentiate between basal cell adenocarcinoma from BCA is the presence or absence of local infiltrative to the adjacent structure. In our case, the tumor was round, firm, mobile, confined, not attached to the skin with intact facial nerve and adjacent nerves such as auriculotemporal nerve and great auricular nerve, lymph nodes and vasculature with no compressive or infiltrative symptoms. There were no skin ulcers or palpable lymph nodes, and the rest of the head and neck evaluation was unremarkable which goes more with BCA rather than basal cell adenocarcinoma [10]. The most popular method of treating BCA is by surgical excision. It is recommended to remove the entire capsule due to the higher risk of both malignant and membranous types to return [20]. To confirm clinical diagnosis, ultrasound-guided fine needle aspiration cytology and magnetic resonance imaging (MRI) of the head and neck were done prior to surgery in the present case. The results of the MRI revealed the tumor was enclosed in a capsule with no invasion to the surrounding structures. Left buccal mass excision was done through transoral approach with a clean wound and intact facial and adjacent nerves such as auriculotemporal nerve and great auricular nerve was observed after full cranial nerves examination. After the surgery there were no complications, and the patient was sent home one day after the surgery in a good, stable condition. She was kept on routine clinic follow-ups, and the surgical site completely healed.

## Conclusions

BCA of a minor salivary gland is a rare benign head and neck tumor. MRI and biopsy provide useful information to differentiate between benign adenoma and malignant adenocarcinoma. BCA should be considered in a differential diagnosis of an isolated neck mass. Surgical excision demonstrates an excellent prognosis.
